# GESC-YOLO: Improved Lightweight Printed Circuit Board Defect Detection Based Algorithm

**DOI:** 10.3390/s25103052

**Published:** 2025-05-12

**Authors:** Xiangqiang Kong, Guangmin Liu, Yanchen Gao

**Affiliations:** 1 School of Railway Transportation, Shandong Jiaotong University, Jinan 250357, China; 23221008@stu.sdjtu.edu.cn; 2 Qingdao Academy of Intelligent Industries, Qingdao 266100, China; gyc@wanlongsys.com

**Keywords:** printed circuit boards, lightweight model, coordinate attention, GSConv, Ghost, defect detection

## Abstract

Printed circuit boards (PCBs) are an indispensable part of electronic products, and their quality is crucial to the operational integrity and functional reliability of these products. Currently, existing PCB defect detection models are beset with issues such as excessive model size and parameter complexity, rendering them ill-equipped to meet the requirements for lightweight deployment on mobile devices. To address this challenge, this paper proposes a lightweight detection model, GESC-YOLO, developed through modifications to the YOLOv8n architecture. First, a new lightweight module, C2f-GE, is designed to replace the C2f module of the backbone network, which effectively reduces the computational parameters, and at the same time increases the number of channels of the feature map to enhance the feature extraction capability of the model. Second, the neck network employs the lightweight hybrid convolution GSConv. By integrating it with the VoV-GSCSP module, the Slim-neck structure is constructed. This approach not only guarantees detection precision but also enables model lightweighting and a reduction in the number of parameters. Finally, the coordinate attention is introduced into the neck network to decompose the channel attention and aggregate the features, which can effectively retain the spatial information and thus improve the detection and localization accuracy of tiny defects (defect area less than 1% of total image area) in PCB defect images. Experimental results demonstrate that, in contrast to the original YOLOv8n model, the GESC-YOLO algorithm boosts the mean Average Precision (mAP) of PCB surface defects by 0.4%, reaching 99%. Simultaneously, the model size is reduced by 25.4%, the parameter count is cut down by 28.6%, and the computational resource consumption is reduced by 26.8%. This successfully achieves the harmonization of detection precision and model lightweighting.

## 1. Introduction

Printed circuit boards (PCBs) are an integral and indispensable component of electronic equipment, playing an exceptionally critical role in modern electronic technology. Their quality directly impacts the performance and reliability of the entire device [1]. Nonetheless, within the context of the PCB production process, due to complex manufacturing processes, external environmental disturbances, and operating errors, PCBs may have surface defects [2] such as short circuits, missing holes, burrs, etc., which affect the normal use of electronic devices. Therefore, effective PCB defect detection is a critical and necessary step in ensuring that electronic equipment performs well and operates reliably.

In the current era of surging digitalization, technological development is undergoing rapid transformation. At the core of this transformation is deep learning, a field of artificial intelligence that is rapidly reshaping numerous industry patterns. In recent years, deep learning has achieved significant breakthroughs in the domain of computer vision [3], especially in image recognition [4], object detection [5], and semantic segmentation. It has shown strong application potential and provides new ideas and methods for PCB defect detection. PCB defect detection methods based on deep learning can automatically mine and learn rich feature expressions [6] from massive data, accurately identify and locate various types of defects on PCBs, significantly improve the accuracy and efficiency of detection, and promote the PCB inspection process towards intelligence and efficiency.

Classical deep learning models have played a crucial role in the development of PCB defect detection. AlexNet is the pioneer of modern deep learning-based image classification techniques, and was the first to demonstrate the power of deep neural networks in processing large-scale image data [7]. Its success has inspired researchers to explore the application of deep learning in PCB defect detection. By utilizing its convolutional layer for feature extraction, it can identify some simple PCB defects to a certain extent. However, due to its relatively shallow architecture, it faces challenges in capturing complex defect features.

Subsequently, VGG was proposed to significantly deepen the network architecture [8]. VGG’s convolutional layer stack has a small-size kernel, which allows for the extraction of more hierarchical and detailed features. In PCB defect detection, this allows for better identification of defects with complex shapes and textures. However, the large number of parameters in VGG leads to high computational cost and potential overfitting problems.

To solve the problem of deep network training, ResNet introduces residual structures [9]. This innovation makes the training of ultra-deep networks independent of the gradient vanishing problem. In PCB defect detection, ResNet’s deep structure captures long-range dependencies and subtle defect features, greatly improving detection accuracy. In addition, its residual connectivity makes the model more robust and easier to train.

Over time, these classical models have been further optimized and adapted specifically for PCB defect detection. For example, the ensemble layer was modified to better preserve the spatial information associated with defect localization. The loss function was also adapted to better suit the characteristics of PCB defect datasets, e.g., to deal with class imbalance. These adjustments improve the performance of the classical deep learning-based PCB defect detection methods and lay a solid foundation for the subsequent development of more advanced detection algorithms.

Contemporary object detection architectures predominantly bifurcate into two methodological paradigms. The first category encompasses two-stage detectors [10], exemplified by Faster RCNN frameworks. Recent advancements in this domain include Hu et al. [11], who used a multi-scale feature re-identification technique based on Faster RCNN to identify defect optimized variants of PCB micro-defects, achieving a mAP of 94.2% in an industrial inspection scenario. Parallel developments by Ding et al. [12] introduced TDD-Net, which integrates pyramid feature distillation with depthwise separable convolutions, establishing new benchmarks of 98.90% mAP on defect datasets. The second paradigm adopts single-stage detection mechanisms, prioritizing computational efficiency through unified regression pipelines. Notable implementations span SSD derivatives and YOLO evolutionary architectures. Wang et al. [13] engineered a YOLOv3-based solder joint analyzer incorporating probability-density weighted anchor optimization, hybrid SE-CBAM attention fusion, and Focal-EIoU loss adaptation, achieving 96.69% mAP with 23 ms inference latency. Subsequent refinements by Liao et al. [14] rearchitected YOLOv4’s backbone with Mish activation and Ghost modules, attaining 98.64% mAP alongside 41% parameter reduction. The YOLO architectural evolution continues with Lim’s [15] YOLOv5 modification employing spatial-frequency attention in FPN pathways, effectively mitigating small-target feature dissipation, with 99% mAP in the experimental results. Chen’s team [16] later proposed a YOLOv7 variant combining a FasterNet backbone with CBAM-enhanced neck structures, resolving the accuracy-efficiency tradeoff in high-throughput PCB inspection and achieving 99.2% mAP at 38 FPS. Most recently, Zhang et al. [17] developed SF-YOLO through YOLOv8’s inspection head miniaturization, demonstrating 0.6% mAP@0.5 improvement with simultaneous 14.2% model compression and 17.2% parametric simplification.

Although significant advancements have been achieved in PCB defect detection research, persistent limitations in computational efficiency and generalizability to real-world industrial scenarios remain unresolved. Conventional CNN architectures, while achieving competitive detection precision, encounter deployment challenges in resource-limited industrial environments due to their substantial parameter sizes and intensive computational requirements. This constraint becomes particularly critical for embedded systems and mobile inspection terminals where hardware resources are strictly constrained. Existing improvements for the YOLO model introduce too many complex mechanisms to increase the detection precision, increase the network complexity and the number of parameters, and make sacrifices in computational cost. The objective is to further decrease the number of model parameters and resource consumption, all the while maintaining a high-precision detection rate for PCB defects. In particular, when it comes to small and intricate PCB surface defects, existing approaches face challenges in simultaneously taking into account detection precision, the quantity of model parameters, and the amount of model memory utilized.

Among the many models, it is crucial to choose the right base model for improvement, and the YOLO series of models have become our focus due to their excellent performance in the field of target detection. Compared with the previous YOLOv5/6/7 versions, YOLOv8n has been optimized in terms of model structure, with lower computational complexity, higher detection accuracy, and improved detection of small targets. Compared to other alternative models, such as EfficientDet, RetinaNet, FCOS, etc., YOLOv8n has a simpler structural design, which makes it easier to customize and improve it for PCB defect detection tasks. For example, its unique module can quickly aggregate multi-scale features, which is highly suitable for PCB defects of different sizes. At the same time, YOLOv8n requires fewer computational resources to process images of the same resolution, making it more suitable for resource-constrained PCB inspection equipment. Combining these advantages, we choose YOLOv8n as the basis for improvement and propose the lightweight inspection algorithm GESC-YOLO, which aims to further optimize the model performance and reduce the number of model parameters and resource consumption while ensuring the inspection accuracy. GESC-YOLO is improved in the following three main aspects:

1. In the GESC-YOLO backbone network, a new lightweight module C2f-GE is designed to replace the original C2f module. This replacement offers several advantages. First, it effectively reduces computational complexity, enhancing the model’s efficiency in processing images and reducing its consumption of computational resources. Second, it causes an increase in the number of channels in the feature map, thereby improving the model’s capacity to recognize diverse types of PCB defects and enhancing its accuracy and generalization capability, ensuring successful performance when confronted with a range of complex PCB defect images.

2. For the GESC-YOLO neck network, we construct a Slim-neck structure using a lightweight hybrid convolutional GSConv in combination with the VoV-GSCSP module. This structural design effectively augments the model’s discriminative capacity, facilitating precise identification of both micro-scale anomalies in printed circuit board inspection imagery. In addition, it successfully solves the problems of an excessive number of model parameters and a too-large model size, making the model more deployable and practical while maintaining high performance.

3. For the uniqueness of PCB defect images, coordinate attention is introduced in the neck network of GESC-YOLO. After the richer defect features are extracted from the previous C2f_GE module, CA can help the model to locate these features in the image more accurately, while the Slim-neck structure ensures that the model is lightweight while providing CA with a more effective representation of the features, which jointly improves the model’s detection and the localization accuracy of small defects. In summary, CA significantly improves the detection and localization accuracy of small defects. By modeling the spatial location information of the features more finely, the model can accurately locate them, thus effectively improving the detection performance. Even for small but complex PCB surface defects, GESC-YOLO ensures that the lightweight model’s detection performance is not compromised by its coordinate focus.

## 2. Materials and Methods

### 2.1. YOLOv8n Algorithm

**Figure 1:**
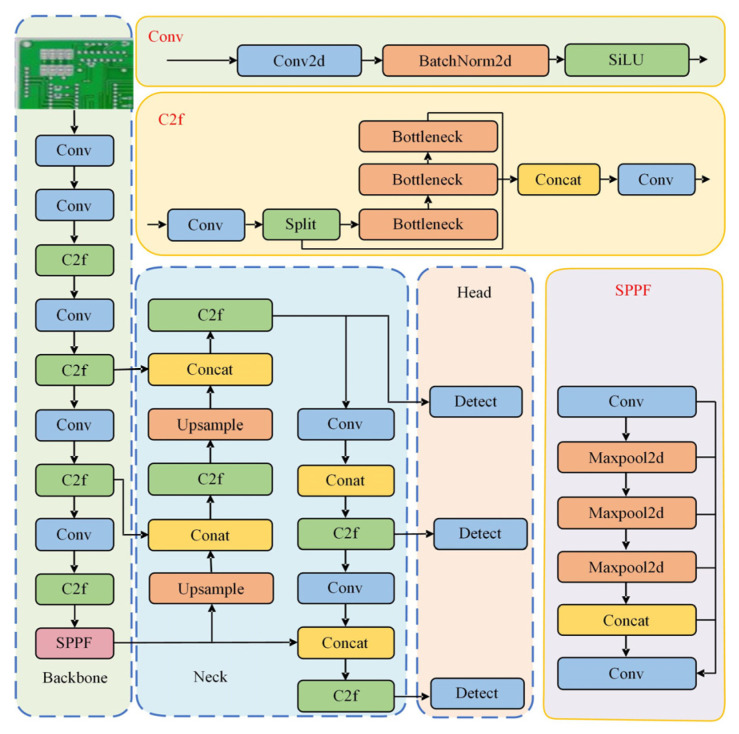
YOLOv8 structure.

YOLOv8 [18] is an object recognition model launched by Ultralytics in 2023. It is a single-stage detection model generated through some unique improvements and performance optimizations based on YOLOv5 and has strong detection capabilities in object detection. Its network structure is presented in Figure 1.

The network structure of YOLOv8 mainly consists of the input, backbone network, neck network, and detection head [19]. The input side is tasked with the preliminary processing of the original image, employing a fundamental input dimension of 640 × 640 pixels to furnish consistent image data for the subsequent network processing. The backbone network is accountable for extracting key features from the input image to provide the necessary information for the subsequent network layers. These layers consist of a convolutional layer (Conv) [20], a composite layer (CSP Darknet 53 to 2 Stage FPN, C2f) [21], and a spatial pyramid pooling fast module (SPPF) [22]. Conv performs feature extraction on the input image and recognizes different features in the image by performing convolutional operations with the input image through a convolutional kernel. C2f consists of a combination of multiple convolutional layers and other operations, which can extract and integrate the features more efficiently and enhance the model’s ability to represent different scales and types of features. SPPF performs multiscale pooling operations on features in different regions [23], thus aggregating feature information under different sensory fields, realizing the combination of local and global features, thereby augmenting the model’s capacity to detect targets of diverse sizes.

The primary function of the neck is to integrate diverse levels of features extracted from the backbone, thereby enhancing the detection capability of targets across various scales. The generation of multi-scale feature maps is achieved through upsampling, downsampling, and other operations, thus catering to the detection requirements of targets of different sizes. At the same time, enhancing feature expression provides higher quality features for subsequent detection heads, improving the accuracy of object detection and localization.

The head is mainly responsible for target detection and recognition. It receives the feature map output from the neck and processes the features using convolution and other operations. On the one hand, it classifies the target and determines the category to which the target belongs. On the other hand, predicting the position and size of the bounding box of the target enables precise localization of the target. The detection head facilitates the model’s output of specific detection results, thereby completing the task of detecting targets in the image.

### 2.2. Improve YOLOv8n Algorithm

#### 2.2.1. GESC-YOLO Model

**Figure 2:**
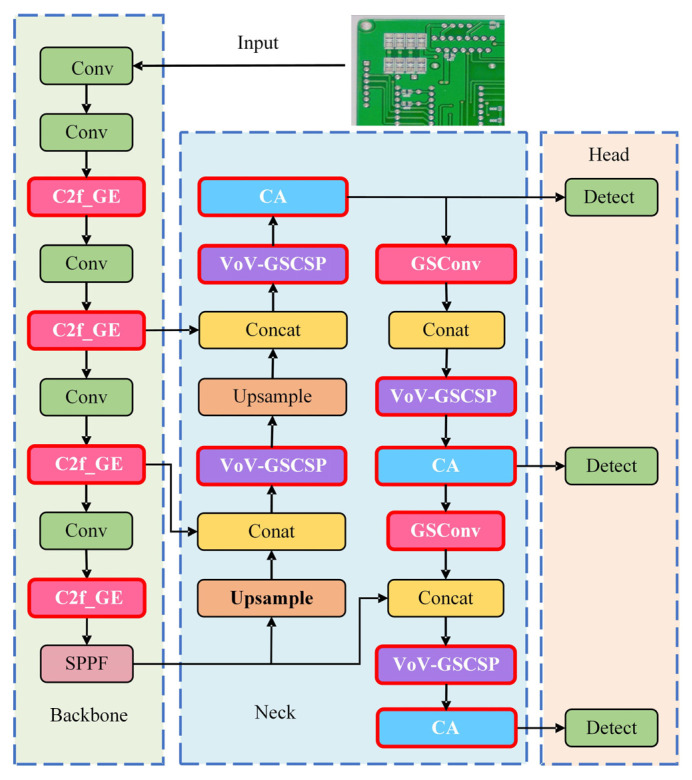
GESC-YOLO model structure.

The GESC-YOLO model has been improved in the following three aspects, and its network structure is presented in Figure 2. All improvement modules are marked with a red border and white font.

First, the lightweight module C2f-GE is designed to replace the original C2f module in the backbone network, which reduces model computation and the number of parameters and increases the number of feature map channels to improve the model accuracy and generalization ability.

Second, the Slim-neck structure is constructed by combining the lightweight hybrid convolutional GSConv and VOV-GSCSP in the neck network. This enables the fusion of feature maps at different levels in the backbone network, the assignment of adaptive weights to the feature maps at different levels, the improvement of the feature extraction ability of the model, the optimization of the network structure, the reduction of the model complexity, and the further reduction of the computation and parameter count of the model during the feature fusion process. This reduction in computation and parameter count is crucial for enhancing the model’s lightweight nature, making it more efficient and practical for various applications.

Finally, the coordinate attention is incorporated into the neck network to accurately capture position and direction information, enhancing the accuracy of detecting and locating small defects on the PCB and ensuring the detection efficiency of the lightweight model. The rich defect features initially extracted by the C2f_GE module can be accurately located by CA, and the Slim-neck structure provides a more discriminative and efficiently fused feature representation for CA, which works closely with the C2f_GE module and the Slim-neck structure to maximize the defect detection performance of the lightweight model.

#### 2.2.2. Backbone C2f_GE Module

The backbone network of YOLOv8 uses C2f modules, mainly composed of two convolutional layers and multiple Bottleneck modules. Its convolution operation and iterative calculation of Bottleneck modules result in a significant increase in computational complexity when processing high-resolution images or large datasets, and there is redundant computation in simple tasks, which slows down training and inference speed [24]. Therefore, this study combines the ideas of GhostNet and EMA (Efficient Multi-scale Attention) to design a new lightweight C2f_GE module, which can effectively reduce computational complexity, increase the number of feature map channels, enrich feature representation, reduce computational costs, enhance feature extraction, make the model focus more on important features, reduce noise impact, and improve model accuracy and generalization ability.

**Figure 3:**
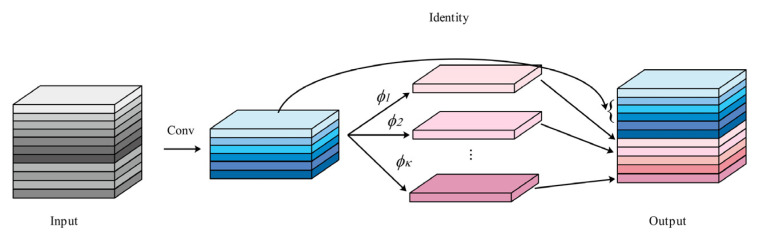
GhostNet Design Core.

GhostNet is a lightweight network [25], with its design core shown in Figure 3. GhostConv is a core module in the GhostNet network that acts as an alternative to ordinary convolution.

To describe the computation complexity of standard convolution operations in neural networks, we consider the input feature map dimensions as X=c×h×w, where c represents input channels and h and w indicate spatial dimensions. The corresponding output Y=c×h’×w’ contains n output channels with modified spatial resolution $h^{’}\times w’$. For convolutional kernels of size k×k, the computational cost $C_{1}$ for conventional convolution can be mathematically expressed as:

(1) $$C_{1}=n\times h’\times w’\times c\times k\times k$$

Let the number of linear operations be s, and the size of the linear operation convolution kernel be d×d. The GhostConv computation $C_{g}$ is:

(2) $$C_{g}=\frac{c\times k\times k\times n\times h’\times w’}{s}+\left(s-1\right)\times \frac{d\times d\times n\times h’\times w’}{s}$$

The ratio of regular convolution to GhostConv computation $r_{s}$ is:

(3) $$r_{s}=\frac{c\times k\times k}{\frac{1}{s}\times c\times k\times k+\frac{s-1}{s}\times d\times d}\approx \frac{s\times c}{s+c-1}\approx s$$

where k is similar to d, and s≪c. The result is approximately equal to s, so GhostConv is more lightweight than conventional convolution.

GhostNet uses a unique GhostConv to extract features with a small number of regular convolutions, then uses simple linear transformations to obtain more information. Finally, it uses concat operations to obtain feature maps that are equivalent in number to regular convolution operations, while maintaining the output feature map size and channel size. This significantly reduces model parameters and computational complexity while maintaining detection performance.

**Figure 4:**
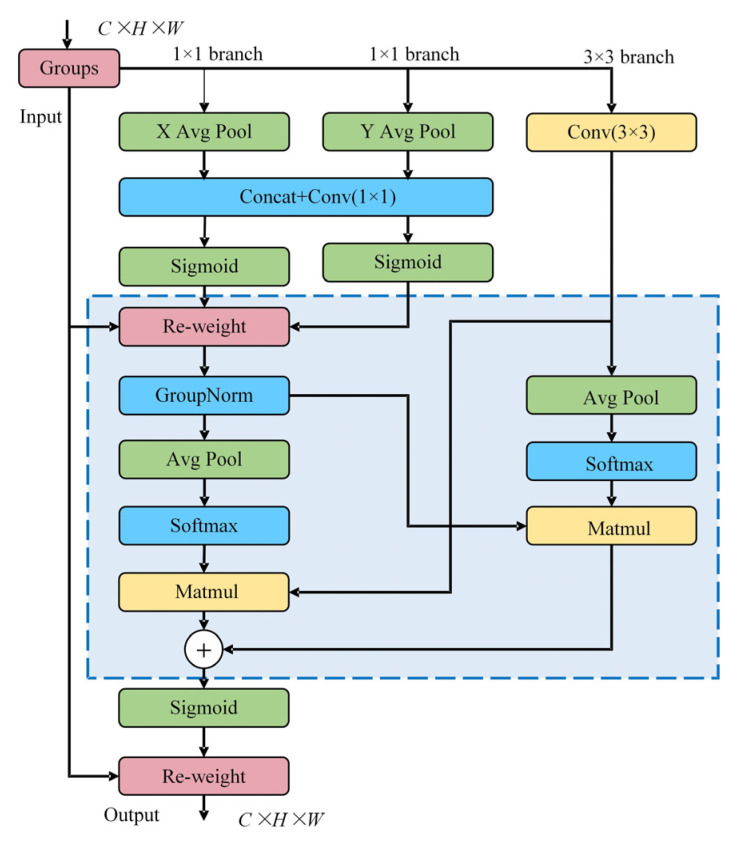
Structure of the EMA.

The EMA [26] module establishes an innovative attention paradigm through cross-spatial interaction mechanisms, employing channel segmentation and dimensional reorganization strategies to optimize feature representation. This architecture enhances discriminative feature perception by implementing parallel multi-scale convolutional operations coupled with cross-space information fusion, thereby improving the network’s ability to identify and emphasize critical feature patterns across different perceptual fields. The hierarchical feature analysis framework effectively captures both local structural details and global contextual relationships through its multi-branch processing workflow. The structure is presented in Figure 4.

First, divide the input feature map into multiple sub feature groups, each containing three parallel branches: two 1 × 1 convolution branches and one 3 × 3 convolution branch. The 1 × 1 convolution branch aggregates the spatial information in the horizontal and vertical directions, respectively, through one-dimensional global average pooling, generating attention maps in these two directions to preserve accurate positional information and avoid spatial information loss that may arise from two-dimensional global pooling. The formula is as follows:

(4) $$Z_{c}^{H}\left(H\right)=\frac{1}{W}\sum_{0\leq i\leq w}x_{C}\left(i,H\right)$$

(5) $$Z_{c}^{H}\left(W\right)=\frac{1}{H}\sum_{0\leq i\leq w}x_{C}\left(j,W\right)$$

where $x_{C}$ denotes the input feature at the c th channel; i,j denote the position of the feature in the horizontal direction W and vertical direction H, respectively; and $Z_{c}^{H},Z_{c}^{W}$ are the global average pooled sh outputs.

The 3 × 3 convolution support utilizes a single 3 × 3 convolution kernel to capture multi-scale features and enhance the feature expression ability of the module. The formula is as follows:

(6) $$Z_{c}=\frac{1}{H\times W}\sum_{i}^{H}\sum_{j}^{H}x_{c}\left(i,j\right)$$

where $\left(i,j\right)$ denotes the location where the feature is located and $Z_{c}$ denotes the 2D global average pooling output.

The EMA module constructs a cross spatial learning network that integrates the outputs of two branches. The network processes the outputs of the 1 × 1 and 3 × 3 branches through global pooling, dot multiplication, and Sigmoid activation functions to generate feature maps with multi-scale spatial information and accurate spatial location information. After adding these two feature maps, they are dot multiplied with the original input features to obtain enhanced features with cross channel and cross spatial information.

The structure of the EMA module enables it to simultaneously encode cross channel feature information and preserve accurate spatial structural information. By using feature grouping and multi-scale processing, we can effectively establish short-range and long-range dependency relationships and further explore the semantic and spatial feature information of the feature map. Its computational cost is minimal, and it can significantly boost the model’s capacity to extract key features without altering the dimensions of the input image, ensuring model performance optimization.

**Figure 5:**
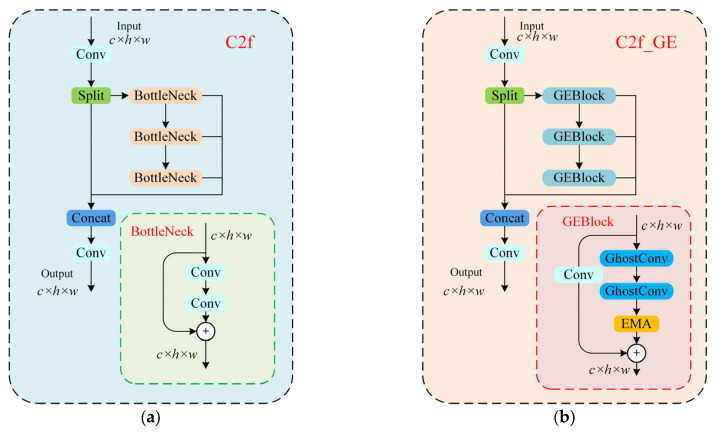
(**a**) Original C2f structure (**b**) C2f_GE structure.

This study integrates GhostConv and EMA into the Bottleneck of C2f to obtain GEBlock, and forms a new lightweight C2f_GE module, whose structure is presented in Figure 5.

PCBs exhibit a diverse range of defect types, each with distinct characteristics. GhostConv is able to generate rich feature maps while reducing the amount of computation, and EMA is able to direct the model to prioritize key defect areas in the PCB image. The combination of these two factors enables the C2f_GE module to more accurately capture the subtle features of various PCB defects. Consequently, this enhances the identification and localization accuracy of different types of defects, while concomitantly reducing the rate of leakage and false detection.

At the same time, PCB images may have complex background interference (e.g., dense line interference) and noise (e.g., pixel anomalies, etc.). The C2f_GE module can theoretically better extract the essential features of defects, thereby enhancing the model’s ability to characterize defects. Among them, GhostConv generates rich feature maps through lightweight operations, which reduces redundant computation while retaining critical information about defects; the cross-space interaction mechanism of EMA guides the model to focus on the defective regions and suppresses the influence of background interference. It significantly reduces false detection and omission caused by external interference, thereby markedly improving the reliability of the detection results. The module design provides theoretical support for modelling such challenges in real industrial scenarios.

The lightweight feature of GhostConv and the reasonable combination with EMA make the C2f_GE module effectively reduce the computational cost and the demand for computational resources under the premise of guaranteeing the detection precision, which realizes the lightweight design requirements.

#### 2.2.3. Slim-Neck Structure of Neck

The YOLOv8 architecture employs conventional standard convolutional operations in its neck network design. Progressive network deepening induces computational overhead and parameter volume escalation, which may lead to model redundancy while processing PCB inspection tasks. Such architectural complexity risks compromising detection reliability and generalization capacity, particularly when handling fine-grained defect patterns. This study introduces a structural optimization strategy that maintains detection fidelity while achieving computational economy through architectural simplification. A lightweight mixed convolution GSConv (Generalized-sparse Convolution) is used in the improved model and is combined with VoV-GSCSP modules to construct a lightweight network structure, Slim-neck.

**Figure 6:**
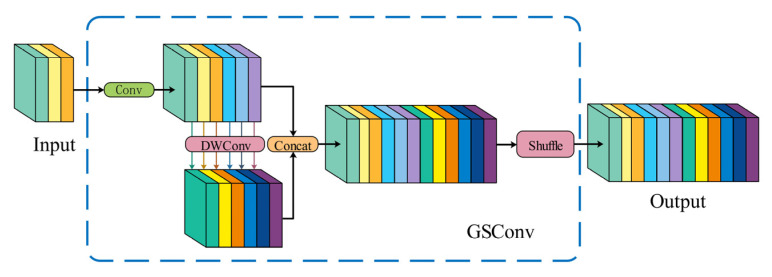
GSConv module structure.

The GSConv structure is presented in Figure 6, which consists of SC, DSC (Depthwise Separable Convolution), and shuffle operations.

GSConv [27] first performs an SC operation on the feature map. Then, a DSC operation is carried out to concatenate the deep semantic information extracted by SC and the shallow feature information extracted by DSC, thus forming a feature map. Finally, it recombines the channel information through a shuffle operation. This method fully utilizes the advantages of two types of convolutions. It can effectively reduce the computational scale and, at the same time, preserve the model’s detection precision.

**Figure 7:**
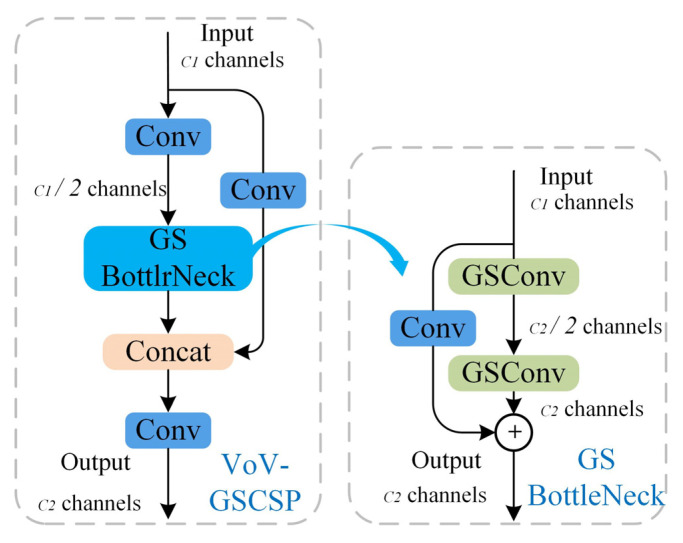
The structures of the VoV-GSCSP module.

VoV-GSCSP [28] is a cross-level partial network module based on the GSBottleNeck module composed of GSConv and Conv. It uses a one-time aggregation method. The VoV-GSCSP module avoids complex calculations, reduces the complexity of the network architecture, and can achieve lightweight design goals without sacrificing accuracy. The specific structure of VoV-GSCSP is shown in Figure 7.

The VoV-GSCSP module has two ways to extract feature information. One method is to directly extract features through ordinary convolution calculation, which is more traditional but concise and effective. It can quickly perform preliminary feature processing on the input feature map and obtain a certain degree of feature information. The other type is more refined. First, it performs regular convolution processing to prepare for the subsequent GSBottleNeck structure designed based on GSConv. The GSBottleNeck structure fully leverages the advantages of GSConv, enabling deeper feature mining and extraction, capturing richer details and semantic information. The feature maps obtained through these two paths are subsequently stitched and output, thereby fusing the features extracted through disparate methods. The VoV-GSCSP module fully utilizes the advantages of GSConv and GSBottleNeck, which enhances the model’s feature extraction ability and simultaneously reduces the model’s parameters. In practical applications, it further improves the lightweight performance of the model while ensuring its accuracy, enabling the model to run efficiently even in resource constrained environments.

**Figure 8:**
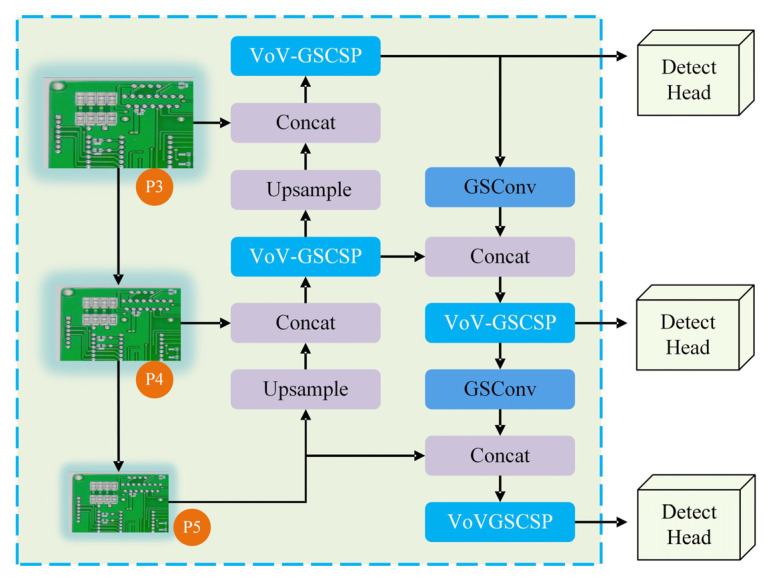
Slim-neck structure in GESC-YOLO.

The lightweight network structure Slim-neck in the GESC-YOLO neck network is shown in Figure 8. Under the premise of guaranteeing the detection precision, the lightweight of the model is further realized.

Regarding its depth, Slim-neck is mainly constructed by GSConv and VoV-GSCSP modules. Among them, the GSConv module performs lightweight hybrid convolutional operations and the VoV-GSCSP module acts as a cross-level partial network module, and the two collaborate with each other. The stacking of these modules is carefully designed in such a way that efficient feature fusion is achieved through information transfer and feature processing between layers

In terms of stage resolution variation, Slim-neck receives feature maps from different layers of the backbone network, which usually have different resolutions, such as P3, P4, and P5. To achieve efficient fusion of feature maps from different layers, operations such as up-sampling and down-sampling are used in Slim-neck. For example, feature maps with P3 resolution will be up-sampled to enhance the resolution to fuse with feature maps with P4 resolution; when fusing with feature maps with P5 resolution, appropriate down-sampling operations may be performed according to the actual situation to ensure the feature maps are compatible in terms of size and semantics, so as to better fuse feature information of different scales.

The proposed Slim-neck architecture strategically optimizes feature propagation efficiency in PCB defect detection through hierarchical feature fusion and cross-scale interaction mechanisms. By integrating GSConv’s dual-path convolution paradigm with VoV-GSCSP’s adaptive aggregation framework, this structure achieves computational economy while maintaining multi-scale representational capacity critical for PCB inspection scenarios.

#### 2.2.4. Coordinate Attention

**Figure 9:**
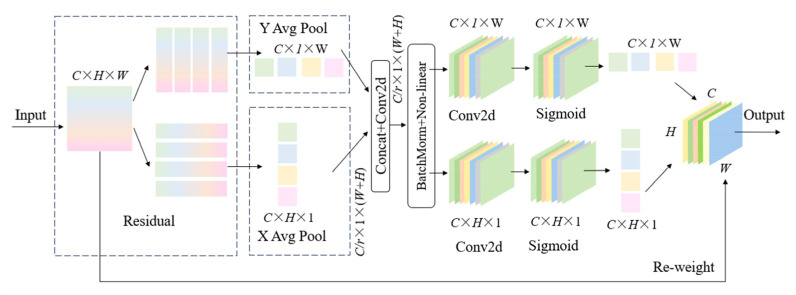
CA Structure Diagram.

In PCB defect images, due to the small pixel size of various defect targets on the PCB surface and their susceptibility to background factors, the YOLOv8 network is prone to feature information loss during feature fusion. Therefore, this paper proposes introducing coordinate attention (CA) [29] into the neck network for feature enhancement, enabling the network to more accurately locate and recognize important information in images. Its structure is presented in Figure 9.

Coordinate attention [30] re-engineers conventional channel attention mechanisms through axis-parallel feature encoding, implementing dual-path processing along vertical and horizontal dimensions.

The activation maps along the width and height dimensions from the global receptive field are concatenated and processed through a 1 × 1 convolutional layer for dimensionality reduction to C/r. The batch-normalized output $F_{1}$ is then activated via the Sigmoid function, generating a feature map of dimensions $C/r\times 1\times \left(W+H\right)$:

(7) $$f=\delta \left(F_{1}\left(\left[z^{h},z^{w}\right]\right)\right)$$

Subsequently, two independent 1 × 1 convolutional operations are applied to f along spatial axes to derive height- and width-oriented feature maps ($F_{h}$ and $F_{w}$, respectively). These maps are normalized using Sigmoid activation to produce attention weights $g^{h}$ and $g^{w}$, respectively:

(8) $$g^{h}=\sigma \left(F_{h}\left(f^{h}\right)\right)$$

(9) $$g^{w}=\sigma \left(F_{w}\left(f^{w}\right)\right)$$

The final attention-weighted feature map y is computed by element-wise multiplication of the original input x with the derived spatial attention weights:

(10) $$y_{c}i,j=x_{c}\left(i,j\right)\times g_{c}^{h}\left(i\right)\times g_{c}^{w}\left(j\right)$$

This architecture synergistically combines orientation-specific feature aggregation to maintain spatial hierarchy while preserving coordinate-level positional accuracy. The mechanism strategically embeds spatial coordinate mapping into channel-wise attention computations through multi-directional feature fusion, enabling expanded receptive fields without computational overhead and thus preserving structural details often compromised by conventional 2D pooling operations.

In the improved model, the Coordinate Attention (CA) mechanism plays a crucial role that is closely intertwined with the previously introduced C2f_GE module and Slim-neck structure. The C2f_GE module, as a key component of the backbone network, has already enhanced the model’s feature extraction capabilities. It effectively reduces computational complexity while increasing the number of feature map channels, which allows it to capture a wide range of PCB defect features, including those of tiny and complex defects. Once the C2f_GE module extracts these rich defect features, CA comes into play. CA’s unique ability to perform axis-parallel feature encoding and dual-path processing enables it to precisely analyze the spatial location information of these features. This means that CA can help the model more accurately locate the defects represented by the features extracted by C2f_GE in the image.

On the other hand, the Slim-neck structure in the neck network is designed with lightweight hybrid convolutional GSConv and VoV-GSCSP modules. This structure not only ensures the model’s lightweight nature but also optimizes the feature fusion process. It effectively combines feature maps at different levels from the backbone network, assigns adaptive weights to these feature maps, and improves the model’s feature extraction ability. In this process, the Slim-neck structure provides CA with more effective feature representations. These refined feature representations allow CA to better focus on the geometric characteristics of defects.

When applied to PCB surface defect detection, this spatially-grounded paradigm of CA, in synergy with C2f_GE and Slim-neck, enhances sensitivity to geometric characteristics of flaws through coordinate-aware feature representation. The enhanced feature extraction by C2f_GE, the optimized feature representation by Slim-neck, and the precise spatial location analysis by CA work together. They enable the model to make full use of spatial information, thereby achieving precise identification and localization of anomalies. This clearly demonstrates the key role of CA in the modular innovation of the GESC-YOLO model, ensuring that the model can achieve high-precision detection while maintaining its lightweight advantage.

## 3. Experimental Platform and Dataset

### 3.1. Introduction to the Experimental Dataset

**Figure 10:**
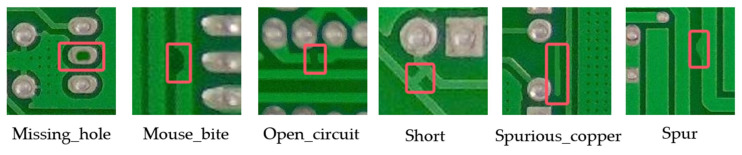
Types of PCB surface defects.

The experimental dataset originated from the PCB defect database publicly released by the Intelligent Robotics Open Laboratory at Peking University, Beijing. The original data were enriched by image enhancement techniques [31], including operations such as rotating, panning, cropping, and adjusting brightness to generate a set of 10,668 enhanced images. The dataset contains six defect types, as shown in Figure 10.

The details of the defective dataset statistics are shown in Table 1. The processed dataset underwent stratified partitioning into training, validation, and testing cohorts, with proportional allocations of 80%, 10%, and 10% respectively.

In this study, define ‘tiny defects’ as defects with an area less than 1% of the total image area. This definition is based on the range of defect sizes commonly found in actual printed circuit board production and the performance of our model in preliminary experiments. This helps us to focus on detecting small-size defects and to evaluate the performance of our model more accurately.

It should be noted that the dataset used in this experiment has specific characteristics. Each image contains only single or multiple instances of a single defect type, and there are no cases where multiple defect types appear in a single image at the same time. This is mainly due to the fact that the data collection process was categorized to more accurately study the model’s ability to detect a single defect type. This approach helps to simplify the research process by focusing on optimizing the model’s performance in identifying and locating specific defects.

In addition, PCB defects are more dispersed throughout the PCB images. There is no obvious aggregation trend between defects by analyzing a large number of images in the dataset. This dispersed distribution simulates the randomness of defects in actual production to some extent and increases the difficulty and challenge of model detection. At the same time, it also provides a good base of data for us to study the detection performance of the model in the case of complex distribution.

### 3.2. Experimental Platform and Model Initialization Configuration

This study used the AutoDL computing power cloud service platform, and the specific configuration is shown in Table 2.

The experimental training parameters for this study are shown in Table 3. The same configuration is used to train, validate, and test all experimental algorithms.

This experiment uses 640 × 640 as the input image size, mainly based on the following considerations:

Matching the characteristics of the dataset: This size closely matches the original size distribution of the PCB images in the dataset, which allows us to make the best use of the image information and avoid the loss or distortion of information caused by size scaling.

Experimental conditions: Combined with the hardware resource limitations of the experimental platform, the 640 × 640 size is able to run efficiently on the GPU (NVIDIA GeForce RTX3080 10G) without the problem of insufficient memory or excessive computational resource consumption.

### 3.3. Experimental Evaluation Metrics

To comprehensively validate GESC-YOLO’s performance, we established a multidimensional evaluation framework incorporating conventional precision, recall metrics, supplemented by mAP (Mean Average Precision), parameters, model size, and FLOPS (Floating-point Operations Per Second) analysis. This systematic approach ensures holistic assessment of both detection accuracy and operational efficiency.

P (Precision) quantifies the reliability of positive class predictions, reflecting the system’s capability to filter out negative samples. Here, TP (True Positive) denotes true positive identifications, while FP (False Positive) represents false alarms from misclassified negative instances. The formula is as follows:

(11) $$P=\frac{TP}{TP+FP}$$

R (Recall) evaluates the model’s coverage of actual positive samples. The FN (False Negative) metric critically indicates system vulnerability to missed detections, particularly crucial for safety-sensitive deployments. The formula is as follows:

(12) $$R=\frac{TP}{TP+FN}$$

For multiclass detection tasks, we implement a hierarchical evaluation protocol: First, category specific AP (Average Precision) is computed, then aggregated across N classes to derive the global performance metric mAP@0.5 (Intersection over Union threshold = 0.5) and mAP@0.5:0.95 (Intersection over Union threshold from 0.5 to 0.95). This approach mitigates evaluation bias caused by category imbalance. The formula is as follows:

(13) $$mAP=\frac{1}{N}\Sigma_{\mathrm{i}=1}^{N}AP_{i}$$

Model complexity is dual-characterized through computational parameters and storage footprint. The parameters reflect the topological complexity of the computational graph, whereas the model size dictates practical storage constraints, jointly determining edge deployment feasibility.

The number of floating point operations per second (FLOPs) is used to evaluate the computational resource consumption of the model. The lower the FLOPS of the model, the lower the consumption of computational resources.

## 4. Experimental Results and Comparative Analysis

### 4.1. Ablation Experiment

To evaluate the optimization efficacy of the lightweight C2f_GE module, this study employs a controlled variable approach within the base model to comparatively analyze the performance of alternative lightweight architectures. The experimental results are shown in Table 4.

According to the analysis of Table 4, replacing the C2f with C2f_Ghost or C2f_Faster can improve the mAP@0.5 of the model and achieve lightweight status, but the precision of the model decreases by 0.4% and 0.1%, respectively, and the mAP@0.5:0.95 of the model decreases by 2.1% and 3.1%, respectively. When using the C2f_EMA module, the model size and parameter count increase while the precision decreases by 0.2%. When using the C2fCIB module, the precision, mAP@0.5, and mAP@0.5:0.95 decrease by 0.3%, 0.1%, and 0.5%, respectively. When using the C2f_GE module designed in this article, the precision and mAP are both improved by 0.1%, and the model size and parameters are reduced by 12.7% and 14%, respectively.

Since the C2f_Ghost module is constructed based on the lightweight idea of GhostNet and generates redundant feature maps through cheap operations, it retains a certain feature expression capability while reducing the amount of convolutional computation, and this design leads to a significant reduction in the number of model parameters. The better performance on the mAP@0.5 metric is because the metric has relatively loose requirements on the overlap between the detection frame and the real frame, and the C2f_Ghost module is able to identify the target effectively with lower precision requirements after quickly extracting the basic features, thus presenting a certain advantage in the mAP@0.5 evaluation.

The C2f_GE module integrates the GhostNet and EMA mechanisms and pays more attention to the effective fusion of multi-scale features and the richness of feature expression in its design. Although the number of parameters is slightly higher than that of C2f_Ghost, the cross-space interaction mechanism of EMA can guide the model to focus on key defective features, and it is far better than C2f_Ghost in mAP@0.5:0.95, which means that the C2f_GE module performs much better in locating and identifying defects, especially in the scenarios that require high detection accuracy. In addition, C2f_GE achieves the lowest level of computational resource consumption (FLOPS), which is 13.4% lower than that of the base model, which indicates that the module achieves a balance between accuracy and efficiency by optimizing the computational process and feature extraction method, which enhances the feature extraction capability while maintaining efficient computation.

The results conclusively demonstrate C2f_GE’s superiority in balancing parameters efficiency and detection precision. This can achieve model lightweighting while ensuring detection precision.

To further verify the effectiveness of each improvement module, a series of ablation experiments were designed and carried out. During the experiments, we adopted two different strategies: one is to add only one improvement module to the base model at a time to observe the effect of the module on the model performance; the other is to try to reasonably combine multiple improvement modules to explore the changes in the model’s performance under different combinations. Through such a comprehensive and detailed experimental setup, the effect of each improvement module and its combination can be precisely analyzed, which provides a strong basis for the optimization of the model. The experimental results are shown in Table 5.

Analyzing the results in Table 5, we can clarify the contribution of each component (C2f_GE, Slim-neck, CA) to the model performance improvement.

C2f_GE module: When the C2f_GE module alone is used to replace the original C2f module, the model precision (P) and mean average precision (mAP) are both improved by 0.1%. At the same time, the model size is reduced by 12.7% (from 6.3 M to 5.5 M), the number of parameters is reduced by 14% (from 3.01 × 10^6^ to 2.59 × 10^6^), and the computational effort (FLOPS) is reduced by 13.4% (from 8.2 G to 7.1 G). This shows that the C2f_GE module plays an important role in the backbone network. This module combines the ideas of GhostNet and EMA, which enables the model to extract key features more efficiently when processing images by reducing the computational complexity. GhostConv generates rich feature maps while reducing the computation, which provides the model with more information that may be used for identifying defects. EMA, on the other hand, performs multi-scale analysis of the input feature maps through its unique cross-space interaction mechanism, guiding the model to focus on critical defect areas in the PCB image. From the experimental results, the accuracy and mAP of the model are improved after using the C2f_GE module. This is because the C2f_GE module increases the number of channels of the feature map and enriches the feature representation, which enables the model to more accurately capture the subtle features of the defect, such as the irregularity of the edge of the defect and the contrast difference with the surrounding area and thus improves the identification and localization of the defect. In conclusion, the C2f_GE module effectively realizes model lightweighting, reduces the consumption of computational resources, improves the operation efficiency of the model, and at the same time improves the detection accuracy of the model.

Slim-neck structure: The Slim-neck structure, which consists of the GSConv and VoV-GSCSP modules, optimizes the feature fusion process in the neck network. GSConv effectively fuses different levels of feature maps through SC, DSC, and shuffle operations, reducing the computational scale while retaining the model’s detection accuracy. The VoV-GSCSP module, on the other hand, fully exploits the feature information through two different feature extraction methods, enhancing the model’s feature extraction capability. When detecting PCB defects, this structure can better integrate the different scales of features output from the backbone network, providing more representative features for the subsequent detection head. When used alone, the Slim-neck structure improves the model accuracy by 0.1% and the mAP@0.5 by 0.2%, reduces the model size by 9.5% (from 6.3 M to 5.7 M), cuts the number of parameters by 10% (from 3.01 × 10^6^ to 2.71 × 10^6^), and reduces the computation amount to 7.1 G. This indicates that the C2f_GE module can ensure the model detection accuracy while reducing the consumption of computational resources. From Table 5, it can be seen that the mAP@0.5 of the model improves to 98.8% after using the Slim-neck structure. For ‘Open-Circuit’ defects, the Slim-neck structure can effectively integrate the information about the line direction and breakpoint features in different layers, enabling the model to more accurately judge whether there is a break in the line and improving the detection capability of these kinds of defects. In summary, the Slim-neck structure further optimizes the model structure and reduces the model complexity on the basis of ensuring the model detection accuracy, achieving a certain degree of lightweighting and improving the model performance.

Coordinate Attention (CA) Module: The Coordinate Attention (CA) Module, when introduced to the neck network, accurately analyses the spatial location information of features through axis-parallel feature encoding and dual-path processing. When detecting PCB defects, CA helps the model to more accurately locate the defects within the image. For instance, in the detection of ‘Mouse-Bite’ defects, CA leverages its unique mechanism to further spatially locate the features of these defects based on the rich features extracted by the C2f_GE module, enabling the model to more precisely identify the defect boundary and location information. When the CA module is introduced alone, it brings about notable improvements in model performance metrics. The precision of the model increases by 0.1%, the recall (R) improves by 0.3%, and mAP@0.5 rises by 0.2%. From the experimental results, these improvements indicate that CA is effective in enhancing the model’s accuracy in detecting and locating defects. In terms of model lightweighting, the model size and the number of parameters remain basically unchanged. This demonstrates that the CA module primarily serves to boost the model’s ability to locate and identify defect features, enhance its detection performance for small defects, and improve the overall detection accuracy without negatively impacting the model’s lightweight nature.

In summary, the C2f_GE, CA, and Slim-neck modules collaborate with each other to improve the model’s ability to reflect PCB defect features from different perspectives: the C2f_GE module enhances the feature extraction capability, the Slim-neck structure optimizes the feature fusion, and the CA improves the defect localization accuracy, which together make the GESC-YOLO model perform well in the PCB defect detection task. The C2f_GE module enhances the feature extraction capability, the Slim-neck structure optimizes the feature fusion, and the CA improves the localization accuracy.

When the three modules are used simultaneously, the accuracy of the model is improved by 0.3%, the mAP reaches 99.0%, which is improved by 0.4%, the size of the model is reduced by 25.4% (from 6.3 M to 4.7 M), the number of parameters is reduced by 28.6% (from 3.01 × 10^6^ to 2.15 × 10^6^), and the computation amount is reduced by 26.8% (from 8.2 G to 6.0 G). The three modules complement each other and play a complementary advantage, which significantly improves the detection accuracy while greatly realizing the light weight of the model and effectively enhancing the comprehensive performance of the model.

### 4.2. Comparative Experiments with Other Benchmark Models

To further substantiate the superiority of the lightweight algorithm GESC-YOLO proposed in this paper in comparison to other algorithms, comparative experiments were conducted, and the results are presented in Table 6.

According to the analysis of Table 6, it can be seen that the mAP of GESC-YOLO is significantly improved, and the model size and parameters are significantly reduced compared to Faster R-CNN and SSD. Compared with the YOLO series of lightweight networks YOLOv5n, YOLOv6n, and YOLOv7-tiny, the improved algorithm GESC-YOLO, while guaranteeing precision, has a mAP@0.5 that increased by 0.3%, 0.5%, and 0.3%, the model size is reduced by 11.3%, 45.9%, and 46.1%, and parameters are reduced by 14.3%, 49.2%, and 64.2%, respectively. Compared with the lightweight model YOLOv8n and large-scale model YOLOv8s of YOLOv8 series, the model size of the improved algorithm is reduced by 25.4% and 79.2%, respectively, the parameters are reduced by 28.6% and 80.6%, and the mAP reaches the highest rate of 99%. Compared with the latest models of YOLO series, YOLOv10n and YOLOv11n, the mAP is improved and the model size decreases by 18.9% and 20.3%, and parameters decrease by 14.5% and 16.7%, respectively. Under the metric of mAP@0.5:0.95, although YOLOv8s is higher than GESC-YOLO, the number of high parameters and resource consumption of the former does not meet the lightweight requirements. GESC-YOLO also reaches the lowest point under the metric of computational resource consumption (FLOPS). This advantage is especially prominent in resource-constrained industrial scenarios, bringing a new breakthrough in the practical application of PCB defect detection technology. It can be concluded that the advantage of the GESC-YOLO algorithm proposed in this paper lies in the significant reduction of the number of parameters, model size, and computational resource consumption while ensuring the detection precision. It performs better in terms of lightness and achieves the highest detection precision with the smallest model size. For resource-limited devices, it can deploy models more simply and efficiently, reducing storage pressure and runtime resource consumption with high efficiency and super-priority.

A comparison of experimental results for various types of defects before and after the improvement is shown in Table 7.

As shown in Table 7 above, GESC-YOLO (ours) significantly outperforms YOLOv8n (base) in several key metrics, demonstrating excellent detection performance. In terms of average precision (AP), GESC-YOLO (ours) achieves an overall AP value of 98.3%, which is higher than the 98.0% of YOLOv8n (base), and achieves accuracy improvement in Missing-Hole, Mouse-Bite, Open-Circuit, and Short categories of detection. Under this mAP@0.5 indicator, GESC-YOLO (ours) outperforms YOLOv8n’s (base) 98.6% with an average value of 99.0%, and the advantage is especially obvious in the Mouse-Bite, Short, and Spurious-Copper categories, which demonstrates that it possesses a more accurate target localization capability at lower intersection-to-parallel ratio (IoU) thresholds.

In a rigorous evaluation of mAP@0.5:0.95, a composite IoU threshold, the average score of GESC-YOLO (ours) of 64.3% is also higher than that of the control model of 63.8%. Although there are slight fluctuations in individual categories, it still maintains the lead in key categories such as Missing-Hole, Mouse-Bite, and Short, which highlights its detection stability and reliability in complex scenes. The above results fully confirm that GESC-YOLO (ours) effectively improves detection accuracy and generalization capability through innovative design and has significant advantages in PCB defect vision inspection tasks.

### 4.3. Visualization and Comparison of Experimental Results

**Figure 11:**
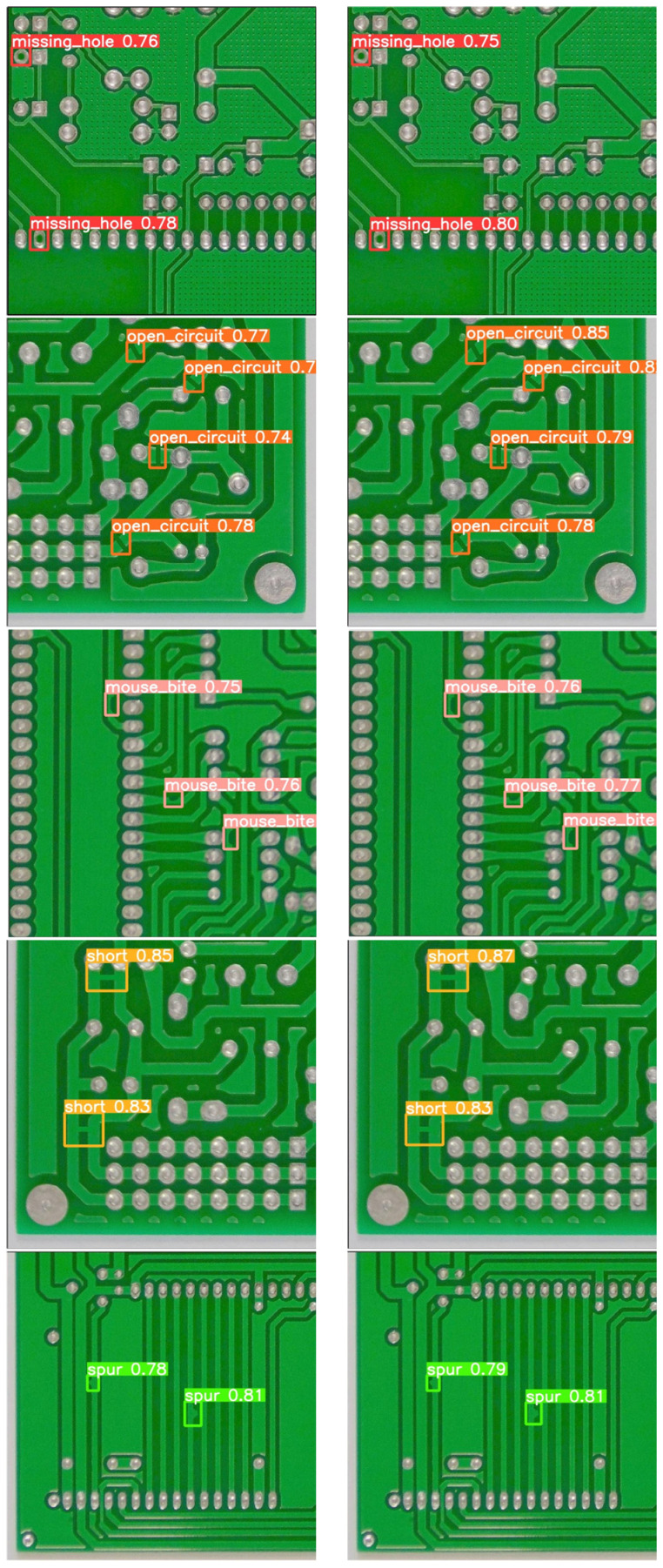

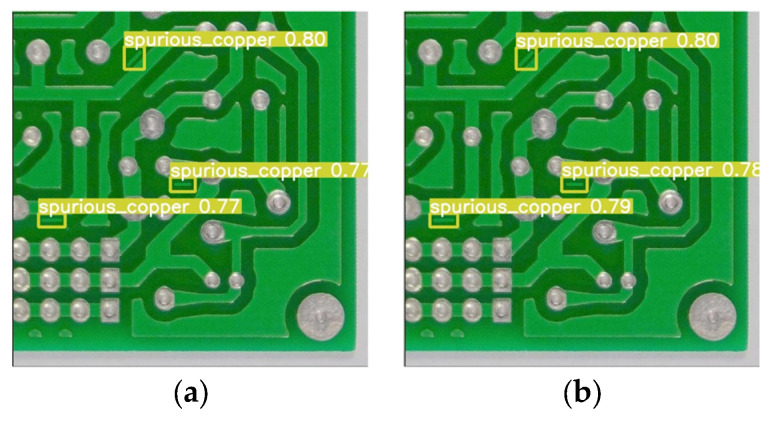
Visual comparison of detection results: (**a**) Original model YOLOv8n detection results, (**b**) Lightweight model GESC-YOLO detection results.

To evaluate the detection efficacy of the proposed GESC-YOLO algorithm, six different defects were selected for comparison with the original YOLOv8n. A comparison of the detection results of the original YOLOv8n and the lightweight model GESC-YOLO is shown in Figure 11.

From the analysis of the detection results in Figure 11, it can be seen that the lightweight model GESC-YOLO in this paper performs extremely well, correctly identified all six types of defects, and there is no problem of false or missed detections. For the detection of mouse_bite and short defects, our algorithm has similar detection performance to YOLOv8n, while for the detection of missing_hole, open_circuit, spurious_copper, and spur, our algorithm has slightly better detection performance than YOLOv8n. The above comparison results show that the GESC-YOLO proposed in this paper ensures and achieves improved detection performance while significantly reducing the number of parameters and the model size and can accurately detect defects on the PCB surface. This means that the lightweight improvement not only does not sacrifice the detection effect, but also exceeds the original model, which fully reflects the great value and effectiveness of lightweight improvement in practical applications.

### 4.4. Limitations and Future Work

Despite the promising results of the GESC-YOLO model in PCB defect detection, it has limitations. When handling non-high contrast defects, the model’s detection accuracy can be compromised as the low contrast makes it hard for the feature extraction modules to precisely identify defect features, potentially leading to missed or misclassified subtle defects. In industrial settings with various noise sources like electromagnetic interference and random pixel noise, the model’s performance suffers since it lacks a dedicated noise suppression mechanism, causing false or missed detections.

Moreover, our current evaluation metrics and model design do not fully account for scenarios where multiple defect types coexist within a single image. This can lead to an inaccurate interpretation of performance metrics such as mAP and recall, especially when dealing with complex defect combinations.

Another limitation is that our study has not comprehensively explored the impact of two important aspects on the model: the spatial resolution dependence and loss function design.

Regarding the spatial resolution dependence, we have not compared the performance of GESC-YOLO and other YOLO versions under different spatial resolutions in this paper. However, understanding the influence of spatial resolution is crucial for optimizing the model’s application. In future work, we will study the performance changes of the GESC-YOLO model at different spatial resolutions to identify the optimal range and improve its adaptability. We will also compare GESC-YOLO with other YOLO versions in terms of spatial resolution utilization to provide a basis for further model improvement.

Concerning loss function design, we used classical and well-established loss functions in this study, such as cross-entropy loss for classification and CIoU loss for regression. The focus of this research was on the impact of model architecture changes on performance, so we did not modify or innovate the loss functions. In future research, we plan to explore loss function design in depth and optimize it in combination with the model architecture proposed in this study to further improve the model’s performance.

For future work, we plan to develop more advanced feature extraction techniques, such as integrating state-of-the-art attention mechanisms or creating novel convolutional layers, to improve sensitivity to low-contrast features. Additionally, we aim to incorporate noise reduction algorithms into the model architecture or apply pre-processing denoising techniques to enhance the model’s robustness against industrial noise. Furthermore, we will focus on optimizing the evaluation strategies for scenarios with multiple defect types per image. This includes exploring more refined metric calculation methods, such as weighted averaging and category-specific evaluations, to better reflect the model’s performance in complex, real-world conditions. These efforts will further optimize the model’s detection performance and facilitate its practical deployment in industrial equipment.

## 5. Conclusions

Aiming at the problem that current printed circuit board defect detection methods cannot balance detection precision and model lightweighting, in this paper, a light-weight detection algorithm, GESC-YOLO, is proposed based on the improved YOLOv8n. First, the novel C2f-GE module is designed to reduce the model computation and enhance the model feature extraction. Then, the neck network adopts the Slim-neck structure constructed by GSConv and combining VoV-GSCSP modules to further reduce the number of parameters and model size. Finally, the coordinate attention is added, and the accuracy of the model for detecting and locating defects on the PCB surface is improved to ensure the detection performance of the lightweight model. The experimental results show that GESC-YOLO shows higher accuracy and mean average precision with fewer number of parameters and smaller model size. The recall of the test set is 98.1%, the precision is 98.3%, and the average precision mean is 99.0%. In terms of model lightweighting, the parameters were reduced by 28.6%, the model size was reduced by 25.4%, and the computational resource consumption by 26.8%. The improved algorithm ensures the detection precision and effectively realizes the model lightweighting. It is of great significance for the application of PCB defect detection. Looking ahead, subsequent research can be centered on further improving the detection performance and efficiency of the model, while actively exploring the practical deployment of the model in industrial equipment. This has a positive application value for improving the automation level in practical scenarios such as industrial quality inspection and helping the electronics manufacturing industry to move towards high-quality development.

## Figures and Tables

**Table 1:** Statistical details of defective datasets.

Defect Types	Images	Defects	Number of Single Defects	Number of Multiple Defects	Defect Size/Pix		
					16 × 16 *	≤32 × 32	≤48 × 48
Missing_hole	1832	3612	716	2896	4	2588	1020
Mouse_bite	1852	3684	700	2984	8	3030	646
Open_circuit	1740	3548	620	2928	412	2949	187
Short	1732	3508	660	2848	2	1479	2027
Spurious_copper	1760	3676	652	3024	0	2087	1589
Spur	1752	3636	572	3064	2	2228	1406
Total	10,668	21,664	3920	17,744	428	14,361	6875

* The minimum defect size is 16 × 16 pix, the image resolution is 96 dpi, and the minimum defect size that can be detected by the model is 4.23 × 4.23 mm.

**Table 2:** Configuration list of experimental platform parameters.

Parameters	Configuration
System	Ubuntu20.0.4
Programming environment	Python3.8
Training framework	PyTorch2.0.0
CPU	Intel(R) Xeon(R) Platinum 8255C
GPU	NVIDIA GeForce RTX3080 10G
CUDA	CUDA 11.8
RAM	40 G

**Table 3:** Experimental model initialization configuration.

Parameters	Configuration
Input size	640 × 640
Scheduler	CosineAnnealingLR
Optimizer	SGD
Weight decay penalties	0.0005
Initial learning rate	0.01
Final learning rate	0.01
Total epochs	300
Training workers	8
Training batch	32
Initial learning rate	0.1
Momentum	0.937

**Table 4:** Lightweight module ablation experiments.

Model	P/%	R/%	mAP@0.5/%	mAP@0.5:0.95/%	Model Size/M	Params/(×10^6^)	FLOPS/G
YOLOv8n	98.0	98.2	98.6	63.8	6.3	3.01	8.2
+C2f_Ghost	97.6	98.3	98.8	61.7	5.5	2.58	7.4
+C2f_Faster	97.9	97.5	98.7	60.7	5.7	2.69	7.4
+C2f_EMA	97.8	98.3	98.8	63.3	6.4	3.05	8.5
+C2fCIB	97.7	98.0	98.5	62.3	5.5	2.56	7.3
+C2f_GE	98.1	98.1	98.7	63.5	5.5	2.59	7.1

**Table 5:** Ablation experiment for each modified module.

C2f_GE	Slim⁃Neck	CA	P/%	R/%	mAP@0.5/%	mAP@0.5:0.95/%	Model Size/M	Params/(×10^6^)	FLOPS/G
			98.0	98.2	98.6	63.8	6.3	3.01	8.2
✔ *			98.1	98.1	98.7	63.5	5.5	2.59	7.1
	✔		98.1	98.2	98.8	64.1	5.7	2.71	7.1
		✔	98.1	98.5	98.8	64.9	6.3	3.01	8.2
✔	✔		97.8	97.9	98.8	61.8	5.0	2.29	6.1
✔		✔	97.7	97.9	98.6	61.5	5.6	2.60	7.2
	✔	✔	97.9	98.1	98.7	64.2	5.7	2.71	7.2
✔	✔	✔	98.3	98.1	99.0	64.3	4.7	2.15	6.0

* Each check mark in Table 5 indicates the use of the corresponding module or structure.

**Table 6:** Comparative experiments between GESC-YOLO and other algorithms.

Model	P/%	R/%	mAP@0.5/%	mAP@0.5:0.95/%	Model Size/M	Params/(×10^6^)	FLOPS/G
Faster RCNN	97.2	92.9	96.3	62.3	178.49	46.76	205.1
SSD	82.7	90.9	88.9	57.5	97.6	25.57	85.7
YOLOv5n	97.8	98.1	98.7	62.4	5.3	2.51	7.1
YOLOv6n	97.4	97.8	98.5	60.8	8.7	4.23	11.9
YOLOv7-tiny	97.7	97.9	98.7	63.2	11.7	6.0	11.7
YOLOv8n(base)	98.0	98.2	98.6	63.8	6.3	3.01	8.2
YOLOv8s	98.3	98.4	98.9	68.9	22.6	11.1	28.6
YOLOv10	97.8	97.7	98.8	63.1	5.8	2.70	7.9
YOLOv11	97.9	98.2	98.9	63.1	5.5	2.58	6.3
GESC-YOLO (ours)	98.3	98.1	99.0	64.3	4.7	2.15	6.0

**Table 7:** Comparison experiments between GESC-YOLO and baseline model YOLOv8n for various defects.

Model	Metric	Missing-Hole	Mouse-Bite	Open-Circuit	Short	Spurious-Copper	Spur	Average
YOLOv8n (base)	AP/%	98.2	98.0	98.3	97.4	97.4	98.5	98.0
	mAP@0.5/%	99.1	98.7	99.1	98.1	98.4	98.3	98.6
	mAP@0.5:0.95/%	68.2	63.6	62.3	63.2	63.4	62.0	63.8
GESC-YOLO (ours)	AP/%	98.4	98.4	98.7	98.1	97.6	98.6	98.3
	mAP@0.5/%	99.2	99.1	99.1	98.3	99.1	98.9	99.0
	mAP@0.5:0.95/%	69.0	64.9	61.3	64.7	64.0	61.8	64.3

## Data Availability

The original dataset used in this article is available from https://robotics.pkusz.edu.cn/resources/dataset/ (accessed on 1 March 2025).
